# Prevalence of peripheral and extra-articular disease in ankylosing spondylitis versus non-radiographic axial spondyloarthritis: a meta-analysis

**DOI:** 10.1186/s13075-016-1093-z

**Published:** 2016-09-01

**Authors:** Janneke J. de Winter, Leonieke J. van Mens, Désirée van der Heijde, Robert Landewé, Dominique L. Baeten

**Affiliations:** 1 Department of Clinical Immunology and Rheumatology, Amsterdam Rheumatology and immunology Center, Academic Medical Center/University of Amsterdam, Meibergdreef 9, 1105 AZ Amsterdam, The Netherlands; 2 Department of Rheumatology, Leiden University Medical Center, Leiden, The Netherlands; 3 Department of Experimental Immunology, Academic Medical Center/University of Amsterdam, Amsterdam, The Netherlands

**Keywords:** Spondyloarthritis, Axial spondyloarthritis, Ankylosing spondylitis, Non-radiographic axial spondyloarthritis, Peripheral manifestations, Extra-articular manifestations, Meta-analysis

## Abstract

**Background:**

Peripheral disease (arthritis, enthesitis and dactylitis) and extra-articular disease (uveitis, psoriasis and inflammatory bowel disease) is common in ankylosing spondylitis (AS) and non-radiographic axial spondyloarthritis (nr-axSpA). So far, however, summary data on the prevalence are lacking. The objective of this meta-analysis was to assess the prevalence of peripheral and extra-articular manifestations in ankylosing spondylitis (AS) and nr-axSpA.

**Methods:**

We performed a systematic literature search to identify publications describing the prevalence of peripheral and extra-articular disease manifestations in patients with AS and nr-axSpA. We assessed the risk of bias and between-study heterogeneity, and extracted data. Pooled prevalence and prevalence differences were calculated.

**Results:**

Eight studies comprising 2236 patients with AS and 1242 with nr-axSpA were included: 7 of the studies were longitudinal cohort studies. There was male predominance in AS (70.4 %, 95 % CI 64.4, 76.0 %) but not in nr-axSpA (46.8 %, 95 % CI 41.7, 51.9), which was independent of the prevalence of human leukocyte antigen (HLA)-B27. The prevalence of HLA-B27 was similar in AS (78.0 % (95 % CI 73.9, 81.9 %) and nr-axSpA (77.4 %, 95 % CI 68.9, 84.9 %)). The pooled prevalence of arthritis (29.7 % (95 % CI 22.4, 37.4 %) versus 27.9 % (95 % CI 16.0, 41.6 %)), enthesitis (28.8 % (95 % CI 2.6, 64.8) versus 35.4 % (95 % CI 6.1, 71.2)). dactylitis (6.0 % (95 % CI 4.7, 7.5 %) versus 6.0 % (95 % CI 1.9, 12.0 %)), psoriasis (10.2 % (95 % CI 7.5, 13.2 %) versus 10.9 % (95 % CI 9.1, 13.0 %)) and inflammatory bowel disease (4.1 % (95 % CI 2.3, 6.5 %) versus 6.4 % (95 % CI 3.6, 9.7 %)) were similar in AS and nr-axSpA. The pooled prevalence of uveitis was higher in AS (23.0 % (95 % CI 19.2, 27.1 %)) than in nr-axSpA (15.9 % (95 % CI 11.8, 20.4 %)).

**Conclusion:**

Peripheral and extra-articular manifestations are equally prevalent in AS and nr-axSpA, except for uveitis, which is slightly more prevalent in AS. These data provide evidence for the largely equal nature of disease manifestations in nr-axSpA and AS.

**Electronic supplementary material:**

The online version of this article (doi:10.1186/s13075-016-1093-z) contains supplementary material, which is available to authorized users.

## Background

Spondyloarthritis (SpA) is a prevalent and potentially disabling form of chronic inflammatory arthritis, affecting 0.5–1.5 % of the Western population [1, 2]. SpA has classically been subdivided into several subtypes, including ankylosing spondylitis (AS), psoriatic arthritis (PsA), reactive arthritis, arthritis/spondylitis associated with inflammatory bowel disease (IBD), and undifferentiated SpA. Classification criteria for SpA have been developed by the Assessment of SpondyloArthritis International Society (ASAS), which classifies SpA as axial or peripheral SpA [3–5]. The axial SpA disease spectrum classifies patients as having either ankylosing spondylitis (AS) whether the modified New York criteria (mNYc) are fulfilled, or as having non-radiographic axial SpA (nr-axSpA) in the absence of definite sacroiliac (SI) joint changes on plain radiograph.

Whether nr-axSpA is a different form [6, 7] of AS, an early form [8–10] of (AS) or two manifestations in the same disease continuum [11–13] is still subject to debate. There are several reasons to assume that AS and nr-axSpA should be considered as the same disease. First, AS and nr-axSpA in general have similar clinical characteristics, especially when related to disease activity [9–11]. Patients with AS and nr-axSpA not only have similar levels of disease activity, they also have a similar clinical disease course in the absence of tumor necrosis factor (TNF)α inhibiting treatment, as shown by recent longitudinal results from the German Spondyloarthritis Inception Cohort (GESPIC) [14]. Second, patients with nr-axSpA respond similarly to TNFα inhibiting treatment [15–18]. Third, radiographic changes only appear after several years; therefore, the requirement for radiographic change clearly reduces the sensitivity of the mNYc. Not only is sensitivity of the mNYc rather limited, but several studies have shown that scoring of radiographs is subject to considerable inter-reader and intra-reader variability. Scoring by both trained readers and local rheumatologists/radiologists not only yields modest sensitivity and specificity at best, but also has only moderate agreement in the recognition of radiographic sacroiliitis [19, 20]. These limitations challenge the crucial role of radiographic scoring in the process of diagnosing AS. Magnetic resonance imaging (MRI) is increasingly being used to visualize inflammation in the SI joints, as active inflammatory lesions are evident on MRI before radiographic lesions are detected [13]. However, MRI also has limitations in terms of scoring agreement, sensitivity, specificity and costs.

On the other hand, AS is characterized by male predominance and a higher level of C-reactive protein (CRP) in comparison to nr-axSpA [9–11]. Other studies suggest that AS and nr-axSpA differ in their genetics, as in some studies human leukocyte antigen (HLA)-B27 carriage is higher in AS than in nr-axSpA [21, 22], whilst other studies suggest there is no difference in HLA-B27 carriage between the two [9, 23, 24].

Even though spinal inflammation and structural damage are the main features of axial SpA, many patients have concomitant peripheral disease (arthritis, enthesitis, dactylitis) and/or extra-articular disease (uveitis, IBD, psoriasis). In AS, previous studies have reported 18–58 % prevalence of arthritis [9, 11, 25–27], 34–74 % prevalence of enthesitis of [9, 25] and a 6–8 % prevalence of dactylitis [9, 26, 28] (reported at any time during the disease course). The reported prevalence of uveitis occurring at some point in time during the course of the disease varies from 22–37 % [25, 27, 29–31], and the prevalence of IBD is estimated at 4–16 % [27, 30–34] and the prevalence of psoriasis at 4–9 % [6, 27, 30, 31, 33, 34].

In contrast, the prevalence of peripheral and extra-articular disease manifestations in nr-axSpA remains less well-defined. Hypothesizing that AS and nr-axSpA reflect subsets of a single disease entity and have similar disease burden, we performed a meta-analysis of published studies of axial SpA in order to assess if the best available estimate of the prevalence of peripheral and extra-articular disease manifestations is similar in AS and nr-axSpA.

## Methods

We conducted a literature search by database searching, citation searching, “pearl growing” [35] and reference list checking. We performed this systematic review and meta-analysis in accordance with the Preferred Reporting Items for Systematic reviews and Meta-Analyses (PRISMA) guidelines [36].

### Search methods

One of the authors (JdW) performed a systematic literature search with the assistance of an experienced librarian (RS). We used the following electronic bibliographical databases: Medline, the Cochrane Central Register of Controlled Trials (CENTRAL) and The Cochrane Library on 1 October 2015 (see Additional file 1: Table S1 for participants, intervention, control and outcome (PICO) and search strategy). The search was performed without language restrictions. In order to retrieve additional references, we used Citation Pearl Growing [35]. Furthermore, primary and secondary references from retrieved publications were manually checked to identify additional studies.

One review author (JdW) screened each title and abstract and selected potentially eligible studies. Thereafter, two review authors (JdW, LvM) independently selected eligible articles according to predetermined selection criteria. If there was any doubt, the full-text article was read by the review authors. Consensus of inclusion was in all cases achieved by discussion. If multiple publications presented data from the same study population, only the publication with the largest sample size was included. Reviews were only included if they presented original data.

### Selection criteria for studies

Studies were included in this meta-analysis if data on prevalence of peripheral and extra-articular disease manifestations in both AS and nr-axSpA were available. We included both longitudinal and cross-sectional studies. Randomized controlled trials and retrospective studies were not included to avoid selection bias and maximize the accuracy of the data. Reporting the prevalence of peripheral and extra-articular disease manifestations did not necessarily have to be the primary outcome of the study.

All patients with axSpA had to fulfill the ASAS axial SpA criteria as defined in 2009 or an equivalent if the study was conducted before 2009 [3] (a modified version of the European Spondylarthropathy Study Group (ESSG) criteria [37]). All patients with AS had to meet the mNY-criteria for AS [38]. All nr-axSpA patients had to fulfill the ASAS MRI criteria or the clinical arm of the ASAS criteria for axial SpA [4] (or an equivalent if the study was conducted before 2009).

### SPondyloArthritis Caught Early (SPACE) cohort study

We added unpublished data to our meta-analysis from one prospective cohort study. The SPACE cohort was described in detail previously [23]. In short, the SPACE cohort is a longitudinal cohort of patients aged 16 years and older, with chronic back pain for at least 3 months but less than 2 years, and with onset before the age of 45 years. In this meta-analysis, we added data for all patients with AS and nr-axSpA included between January 2009 and 2014 at two of the participating centers (Academic Medical Center Amsterdam and the Leiden University Medical Center).

### Data extraction and management

Two review authors (JdW, LvM) independently extracted data using a predesigned form. The following details were extracted whenever available: first author, name of the study, country, year of publication, study design, study characteristics, sample size, mean age of patients, male/female ratio, mean disease duration, HLA-B27 positivity and percentage of the cohort in which HLA-B27 was measured and the timing and prevalence of peripheral and extra-articular disease manifestations. Whenever data on certain peripheral or extra-articular disease manifestations were missing, we contacted the authors of the article concerned. Furthermore, we requested data on the distribution of male/female participants among HLA-B27-positive and HLA-B27-negative patients with AS and nr-axSpA.

### Assessment of risk of bias

Two review authors (JdW, LvM) assessed the potential risk of bias in all of the included studies by using the Methodological evaluation of Observational REsearch (MORE) checklist [39], which we adapted to our research question. The MORE checklist includes parameters on reporting of statement of potential conflicts of interest, study funding, ethical approval, external validity (was the gold standard for the diagnosis of AS and nr-axSpA used? Was there (reporting on) sampling bias?), and internal validity (how and when was the prevalence of peripheral or extra-articular disease symptoms measured?).

### Assessment of heterogeneity

First, we assessed qualitative heterogeneity across studies by comparing them according to predefined criteria. Second, we evaluated the degree of statistical heterogeneity and inconsistency by using the *T*_2_, Chi^2^ and *I*^2^ statistics. Heterogeneity was considered significant at *P* < 0.10 [40]. *I*^2^ values of 25, 50 and 75 % were considered to indicate low, moderate and high inconsistency, respectively [40]. We used a random effects model for studies that we considered sufficiently homogeneous to include in the meta-analysis, as we anticipated there would not be one true effect size for all studies, despite potential measured quantitative homogeneity [41].

### Data synthesis

We pooled studies in a random-effects model using the Mantel-Haenszel method, which estimates the between-study variation by comparing the result of each study with a Mantel-Haenszel random-effect meta-analysis result. Data were calculated as pooled prevalence with the corresponding 95 % confidence interval (CI) and as the difference in pooled prevalence between AS and nr-axSpA, with the corresponding 95 % CI. We performed data analyses using MetaXL [42] in Microsoft Excel 2010 and Review Manager 5. Forest plots were produced for all analyses using Review Manager 5, and we adapted these to our specific needs (to show the difference in prevalence instead of the difference in risk).

## Results

### Search

**Fig. 1:**
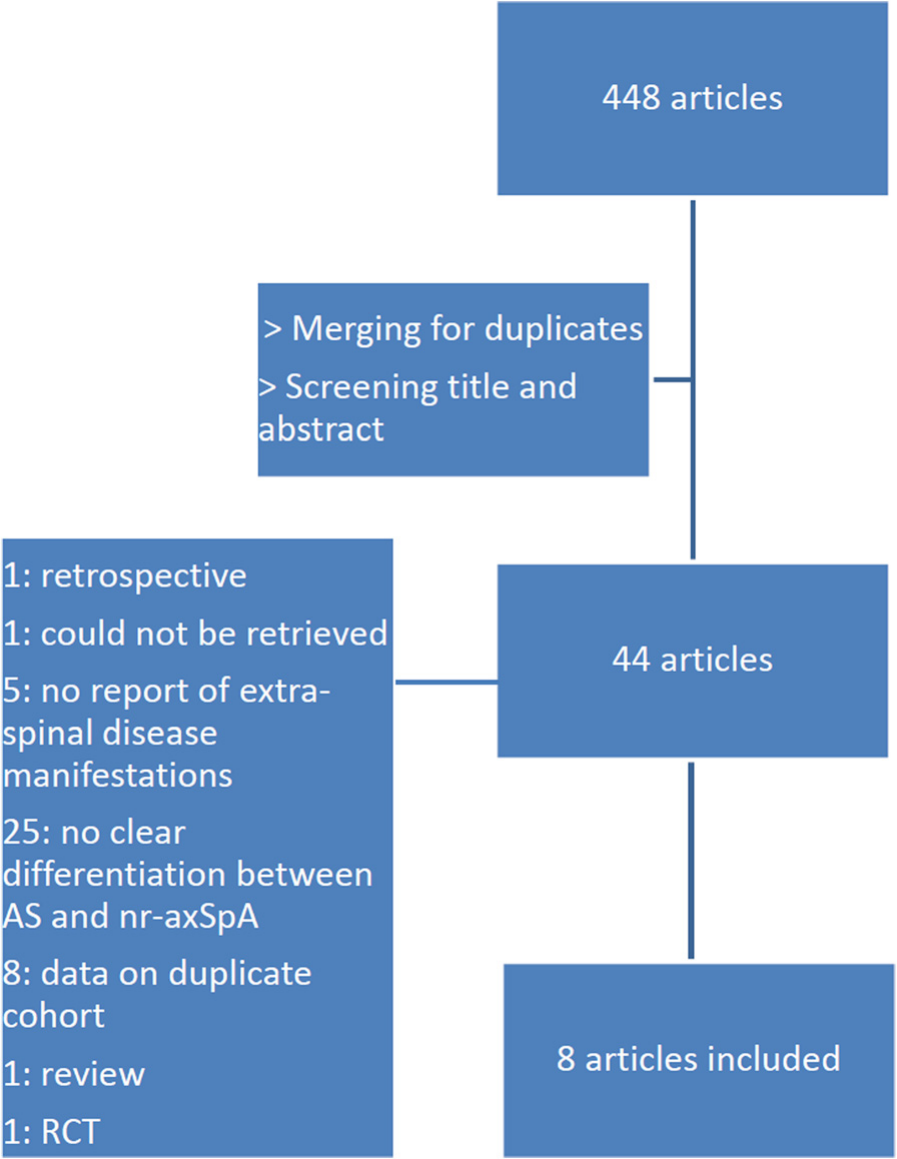
Flow chart of included studies on prevalence of peripheral and extra-articular disease manifestations in non-radiographic axial spondyloarthritis and ankylosing spondylitis

The electronic database search identified 447 articles (Fig. 1). The use of citation pearl growing resulted in one additional article. After merging for duplicates and screening of the titles and abstracts, we completely reviewed 44 unique articles. One article reported retrospective data. One full-text article could not be retrieved. Five articles did not report symptoms of peripheral or extra-articular disease manifestations. In 25 articles, there was no clear differentiation between AS and nr-axSpA. Two articles did not use the ASAS and mNY criteria to classify axial SpA. Eight articles reported data on duplicate cohort studies. Finally, eight studies were included in the meta-analysis [6, 11, 23, 43–45].

### Characteristics of the included studies

Eight studies fulfilled the inclusion criteria. The characteristics of the enrolled studies are summarized in Table 1. Seven out of eight studies were longitudinal SpA cohort studies. One study was a cross-sectional study of 100 consecutive patients attending outpatient clinics. In total, 3478 patients were included, of whom 2236 had AS and 1242 had nr-axSpA. Patients with AS fulfilled the mNY criteria in all studies. Patients with nr-axSpA fulfilled the ASAS criteria in six out of eight studies. Both GESPIC and Swiss Clinical Quality Management (SCQM) used the ASAS criteria with minor modifications, as patients were included before the development of the ASAS criteria.

**Table 1:** Characteristics of the eight included studies.

Name cohort or author	nr-axSpA *n*	AS *n*	Study design	Year of inclusion	Data collection	Maximum disease duration for inclusion, years	Mean symptom duration nr-axSpA, mean years (SD)	Mean symptom duration AS, mean years (SD)
GESPIC	226	236	Cohort	2000–2004	Baseline	AS: <10; nr-axSpA <5	2.6 (1.7)	5.2 (2.3)
Kiltz	44	56	Cross-sectional	Unknown	n.a.	No	9.4 (9.5)	12.8 (10.7)
DESIR	295	180	Cohort	2007–2010	Baseline	<3	1.5 (0.9)	1.6 (0.9)
SCQM	232	838	Cohort	2005–2011	Baseline	No	5.5 (1.8–13.7)^b^	12.7 (6.4–22.7)^b^
SPACE	58	23	Cohort	2009–2014	Baseline	<2	1.1 (0.6)	1.3 (0.7)
Esperanza	182	109	Cohort	2008–2011	Baseline	<2	1.0 (1.6)	1.2 (0.5)
Wallis	73	639	Cohort	2003–2012	Baseline	No	12.1 (8.5)^a^	17.7 (12.3)^a^
ESPAC	132	155	Cohort	2009–2014	Unknown	No	5.8 (5.5)	11.7 (7.7)

^a^Mean disease duration. ^b^Median (interquartile range). *Nr-axSpA* non-radiographic axial spondyloarthritis, *AS* ankylosing spondylitis, *SD* standard deviation, *GESPIC* GErman SPondyloarthritis Inception Cohort, *n.a.* not applicable, *DESIR* Devenir des Spondylarthropathies Indifferenciées Récentes, *SCQM* Swiss Clinical Quality Management, *SPACE* SPondyloArthritis Caught Early, *ESPAC* Erciyes SPondyloArthritis

The mean symptom duration varied from 1.2 ± 0.6 years in AS and 1.0 ± 0.7 year in nr-axSpA (Esperanza) to 17.7 ± 12.3 years in AS and 12.1 ± 8.5 years in nr-axSpA (Wallis). In pooled analysis the male prevalence of AS was 70.4 % (CI 64.4-76.0 %) and of nr-axSpA it was 46.8 % (CI 41.7, 51.9 %), resulting in a difference in pooled prevalence of 23.2 % (CI 15.3, 31.1 %). When we compared the male prevalence of AS and nr-axSpA among HLA-B27-positive and HLA-B27-negative patients, the difference in prevalence remained (24.2 % more male patients among patients with AS who were HLA-B27-positive (CI 15.1, 32.9 %) and 22.3 % more male patients among those with AS who were HLA-B27-negative (CI 14.4, 30.0 %)). The pooled prevalence of HLA-B27 did not differ in AS compared to nr-axSpA, with 78.0 % prevalence in AS (CI 73.9, 81.9 %) in AS compared to 77.4 % (CI 68.9, 84.9 %) in nr-axSpA.

All eight studies collected information about peripheral and extra-articular disease manifestations at baseline. Three out of eight studies (GESPIC, Kiltz and SPACE) reported patients who currently had, or had ever had, peripheral and extra-articular manifestations. Three studies only reported disease manifestations that had ever occurred (SCQM, Wallis and ESPAC) and two cohort studies reported only current disease manifestations (DESIR, Esperanza).

### Risk of bias

All published studies (seven out of eight) were published in journals with an impact factor ˃3.5. The risk of bias summary for all studies is shown in Additional file 2: Table S2. In short, the risk of bias in all studies was considered sufficiently low to be included in this meta-analysis. General bias (role of funding, reporting on ethical approval, conflict of interest) was considered low in all eight studies. Internal validity was considered high in three out of eight studies (Esperanza, SCQM and SPACE) and intermediate in five out of eight studies. The source of the measure of prevalence of peripheral or extra-articular disease manifestations was reported in two of the eight studies (SCQM and SPACE). External validity was considered high in five out of eight studies (Esperanza, Kiltz, SCQM, SPACE and DESIR), intermediate in one out of eight studies (ESPAC) and low in two out of eight studies (GESPIC and Wallis).

### Heterogeneity

When comparing all study characteristics as summarized in Table 1, all eight studies were homogeneous enough to include in the meta-analysis. Statistical heterogeneity was measured for each of the peripheral or extra-articular disease manifestations.

### Meta-analysis

#### Peripheral arthritis

**Fig. 2:**
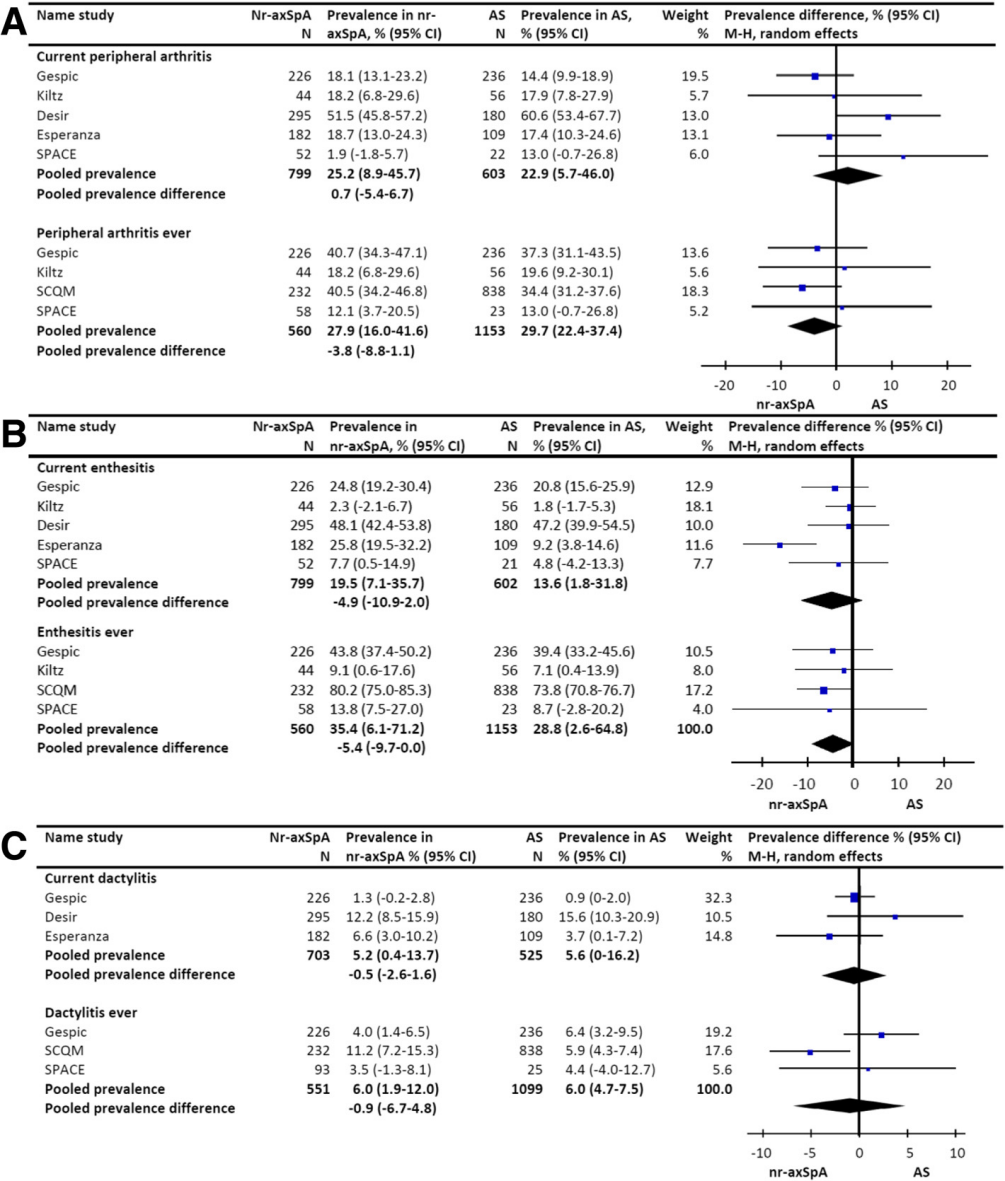
Prevalence of peripheral manifestations in patients with ankylosing spondylitis and non-radiographic axial spondyloarthritis. **a** pooled prevalence difference of arthritis in patients with ankylosing spondylitis versus non-radiographic axial SpA. **b** pooled prevalence difference of enthesitis in patients with ankylosing spondylitis versus non-radiographic axial SpA. **c** pooled prevalence difference of dactylitis in patients with ankylosing spondylitis versus non-radiographic axial SpA. *GESPIC* GErman SPondyloarthritis Inception Cohort, *SCQM* Swiss Clinical Quality Management, *SPACE* SPondyloArthritis Caught Early, *M-H* Mantel-Haenszel

In pooled analysis the prevalence of current peripheral arthritis was 22.9 % (CI 5.7, 46.0 %) in AS and 25.2 % (CI 8.9, 45.7 %) in nr-axSpA, resulting in a difference in pooled prevalence of 0.7 % (CI –5.4, 6.7 %) favoring AS. Quantitative heterogeneity was not statistically significant and the level of inconsistency was moderate (Chi^2^ = 7.29, *P* = 0.12, Tau^2^ = 0.00, *I*^2^ = 45 %). The pooled prevalence of a history of peripheral arthritis was 29.7 % (CI 22.4, 37.4 %) in AS and 27.9 % (CI 16.0, 41.6 %) in nr-axSpA, resulting in a difference in pooled prevalence of –3.8 % (CI –8.8, 1.1 %) favoring nr-axSpA (Fig. 2a). Quantitative heterogeneity was not statistically significant and the level of inconsistency was low (Chi^2^ = 1.25, *P* = 0.74, Tau^2^ = 0.00, *I*^2^ = 0 %).

#### Enthesitis

In pooled analysis the prevalence of current enthesitis was 13.6 % (CI 1.8, 31.8 %) in AS and 19.5 % (CI 7.1, 35.7 %) in nr-axSpA, resulting in a difference in the pooled prevalence of –4.9 % (CI -10.9, 1.0 %) favoring nr-axSpA. Quantitative heterogeneity was statistically significant and the level of inconsistency was moderate (Chi^2^ = 11.37, *P* = 0.02, Tau^2^ = 0.00, *I*^2^ = 65 %). The pooled prevalence of a history of enthesitis was 28.8 % (CI 2.6, 64.8 %) in AS and 35.4 % (CI 6.1, 71.2 %) in nr-axSpA, resulting in a difference in pooled prevalence of –5.4 % (CI -9.7, 0.0 %) favoring nr-axSpA (Fig. 2b). Quantitative heterogeneity was statistically significant and the level of inconsistency was moderate (Chi^2^ = 12.17, *P* = 0.03, Tau^2^ = 0.00, *I*^2^ = 34 %).

#### Dactylitis

In pooled analysis the prevalence of current dactylitis was 5.6 % (CI 0.0, 16.2 %) in AS and 5.2 % (CI 0.4, 13.7 %) in nr-axSpA, resulting in a difference in pooled prevalence of –0.5 % (CI -2.6-1.6 %) favoring nr-axSpA. Quantitative heterogeneity was not statistically significant and the level of inconsistency was moderate (Chi^2^ = 3.31, *P* = 0.19, Tau^2^ = 0.00, *I*^2^ = 40 %). The pooled prevalence of a history of dactylitis was 6.0 % (CI 4.7, 7.5 %) in AS and 6.0 % (CI 1.9, 12.0 %) in nr-axSpA, resulting in a pooled prevalence difference of –0.9 % (CI -6.7, 4.8 %) favoring nr-axSpA (Fig. 2c). Quantitative heterogeneity was statistically significant and the level of inconsistency was moderate (Chi^2^ = 7.37, *P* = 0.03, Tau^2^ = 0.00, *I*^2^ = 73 %).

#### Uveitis

**Fig. 3:**
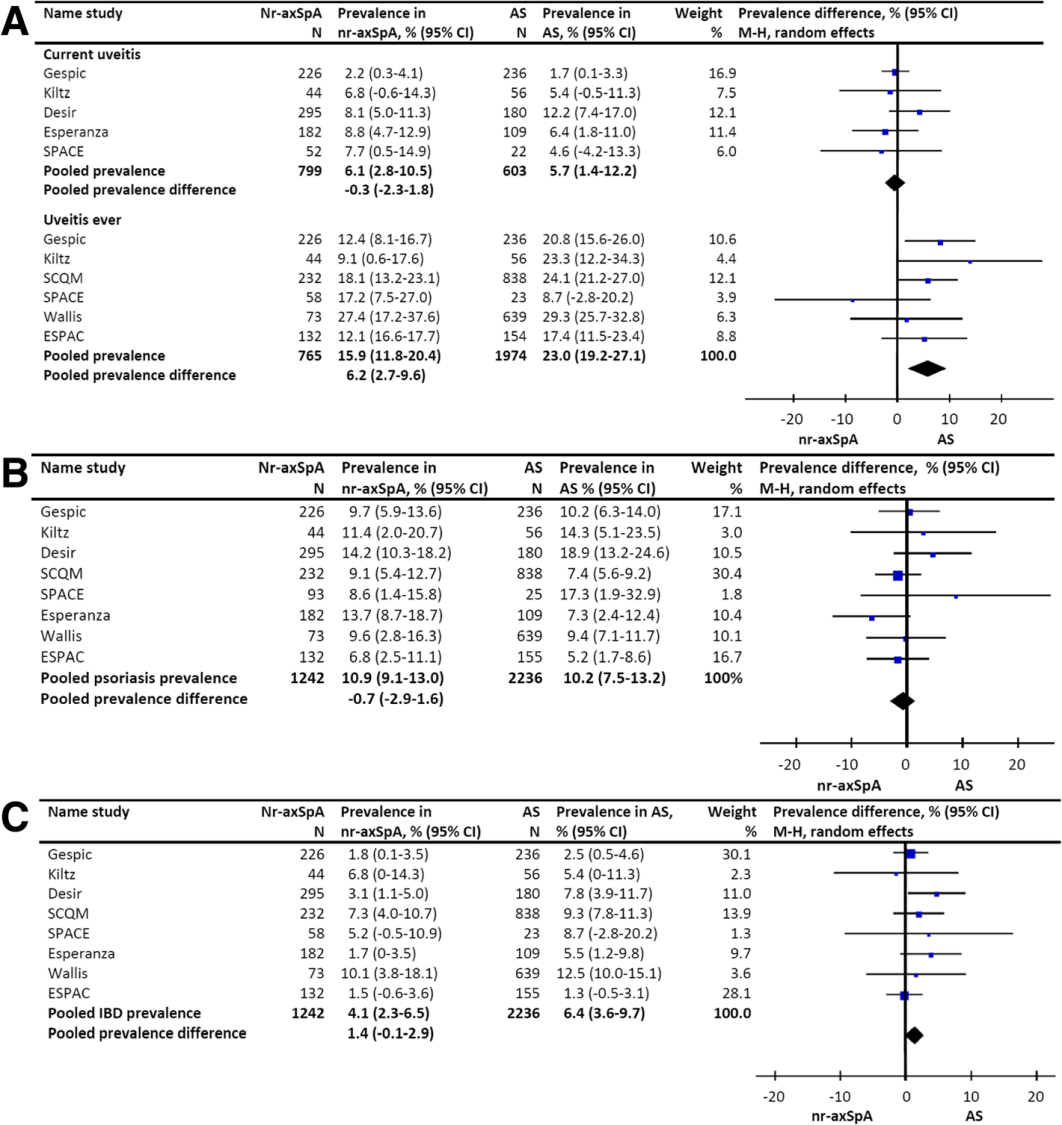
Prevalence of extra-articular manifestations in patients with ankylosing spondylitis and non-radiographic axial spondyloarthritis. **a** pooled prevalence difference of uveitis in patients with ankylosing spondylitis versus non-radiographic axial SpA. **b** pooled prevalence difference of psoriasis in patients with ankylosing spondylitis versus non-radiographic axial SpA. **c** pooled prevalence difference of IBD in patients with ankylosing spondylitis versus non-radiographic axial SpA. *GESPIC* GErman SPondyloarthritis Inception Cohort, *SCQM* Swiss Clinical Quality Management, *SPACE* SPondyloArthritis Caught Early, *M-H* Mantel-Haenszel

In pooled analysis the prevalence of current uveitis prevalence was 5.7 % (CI 1.4-12.2 %) in AS and 6.1 % (CI 2.8-10.5 %) in nr-axSpA, resulting in a difference in pooled prevalence of -0.3 % (CI -2.3-1.8 %) favoring nr-axSpA. Quantitative heterogeneity was not statistically significant and the level of inconsistency was low (Chi^2^ = 3.46, *P* = 0.48, Tau^2^ = 0.00, I^2^ = 0 %). The pooled prevalence of a history of uveitis was 23.0 % (CI 19.2-27.1 %) in AS and 15.9 % (CI 11.8-20.4 %) in nr-axSpA, resulting in a difference in pooled prevalence of 6.2 % (CI 2.7, 9.6 %) favoring AS (Fig. 3a). Quantitative heterogeneity was statistically significant and the level of inconsistency was low (Chi^2^ = 5.94, *P* = 0.008, Tau^2^ = 0.00, *I*^2^ = 16 %).

#### Psoriasis

In pooled analysis the prevalence of psoriasis was 10.2 % (CI 7.5, -13.2 %) in AS and 10.9 % (CI 9.1, 13.0 %) in nr-axSpA, resulting in a difference in pooled prevalence of –0.7 % (CI –2.9, -1.6 %) favoring nr-axSpA (Fig. 3b). Quantitative heterogeneity was not statistically significant and the level of inconsistency was low (Chi^2^ = 7.01, *P* = 0.43, Tau^2^ = 0.00, *I*^2^ = 0 %).

### Inflammatory bowel disease

Pooled analysis showed an IBD prevalence of 4.1 % (CI 2.3-6.5 %) in AS and 6.4 % (CI 3.6-9.7 %) in nr-axSpA, resulting in a pooled prevalence difference of 1.4 % (CI -0.1-2.9 %) favoring AS (Fig. 3c). Quantitative heterogeneity was not statistically significant and the level of inconsistency was low (Chi^2^ = 6.17, *P* = 0.52, Tau^2^ = 0.00, I^2^ = 0 %).

## Discussion

In this meta-analysis we have shown that peripheral disease (arthritis, enthesitis, dactylitis) and extra-articular disease (uveitis, psoriasis, inflammatory bowel disease) manifestations are frequent and, with the exception of a history of uveitis, equally prevalent in AS and nr-axSpA.

Our data on peripheral disease are consistent with earlier published data on peripheral disease in AS, with 18–58 % prevalence of arthritis [9, 11, 25–27], 34–74 % prevalence of enthesitis [9, 25] and 6–8 % prevalence of dactylitis [9, 26, 28] (all reported at any time during the disease). In one study by Vander Cruyssen et al. the prevalence of arthritis (58 %) and enthesitis (50 %) (ever occurrence) was higher [25]. Longer mean symptom duration (11 years) and the retrospective reporting in this study may have caused this difference. Importantly, the prevalence of peripheral disease in AS was not reported as the primary outcome in any of the AS studies.

Our prevalence data for extra-articular disease were also in line with previous data on AS, showing 22–37 % prevalence of uveitis [25, 27, 29–31], 4–16 % prevalence of IBD [27, 30–34], and 4–9 % prevalence of psoriasis [6, 27, 30, 31, 33, 34], which all adds to the robustness of our aggregated estimates. The prevalence of uveitis in our study population was lower than in the meta-analyses of Stolwijk et al. and Zeboulon et al., who reported pooled prevalence of 25.8 % and 32.7 %, respectively [29, 30]. This might be caused by a higher mean disease duration (15.9 years in the meta-analysis of Stolwijk et al. and 17.7 years in the meta-analysis of Zeboulon et al.). Another explanation for this difference in prevalence is that both review studies also included clinical trials, enriched with patients with more active and severe disease.

When we compared patients with AS and nr-axSpA, HLA-B27 was equally prevalent in patients with nr-axSpA and AS. Because HLA-B27 is the main entry requirement for fulfilling the ASAS criteria for nr-axial SpA via the clinical arm, HLA-B27 might be artificially overrepresented in the nr-axSpA arm. However, the Esperanza cohort differentiated axSpA in AS and nr-axSpA in a clinical and imaging arm, enabling us to compare the HLA-B27 prevalence in both arms. There was no statistically significant difference in the prevalence of HLA-B27 in the imaging arm of nr-axSpA (where HLA-B27 is not required to fulfill the ASAS criteria) and in AS (58.3 % vs. 67.6 %, respectively) [44], providing evidence for equal HLA-B27 prevalence in nr-axSpA and AS.

Our data show that AS patients were more frequently male than nr-axSpA patients (23.2 % difference in prevalence). These results are in line with previous studies showing that male patients with axial SpA have more structural damage on radiographs than female patients [11, 46–48]. Because the mNY criteria require radiographic evidence of sacroiliitis, channeling of male patients occurs. For nr-axSpA, this channeling does not exist, which is reflected in the equal gender distribution in nr-axSpA.

Interestingly, when we subcategorized the study population in HLA-B27-positive and HLA-B27-negative patients with AS or nr-axSpA, the male predominance among patients with AS was the same in HLAB27-positive and HLAB27-negative patients, challenging the concept of AS as an HLA-B27-positive driven, predominantly male disease.

Our study data show that peripheral and extra-articular disease manifestations are, with the exception of uveitis, frequent and equally prevalent in AS and nr-axSpA. These data further support the concept of axial SpA being one disease continuum irrespective of the presence and extent of radiographic changes [11–13]. Two important conclusions can be drawn from this meta-analysis. First, peripheral and extra-articular disease manifestations significantly contribute to the burden of disease in axial SpA. These results are in contrast with the relatively limited contribution of peripheral and extra-articular disease to disease monitoring and outcome measurement in axial SpA. Second, these results show that differentiating between AS and nr-axSpA is artificial and should therefore be avoided, especially when selecting patients for research and treatment. These patients can best be combined into one group with axial SpA.

Uveitis is less prevalent in nr-axSpA than in AS (with a difference in the pooled prevalence of 6.2 %). This difference was not explained by a difference between nr-axSpA and AS in the prevalence of HLA-B27, as this did not differ between the two groups. Zeboulon and colleagues showed that the prevalence of uveitis was higher in HLA-B27-positive patients [29], although this was not confirmed by Stolwijk et al. [30] in their meta-analysis of extra-articular manifestations in AS. The higher prevalence of uveitis in AS in this meta-analysis might be explained by the longer mean disease duration (presuming that uveitis in axial spondyloarthritis does not necessarily occur at the start of the disease and thus needs time to develop). This hypothesis is supported by 1) the fact that in the included cohort studies with longer mean disease duration there was generally a higher prevalence of a history of uveitis (Kiltz, SCQM, Wallis): excluding those cohort studies from the meta-analysis resulted in a non-significant difference between AS and nr-axSpA in the prevalence of uveitis (data not shown) and 2) the fact that the prevalence of current uveitis did not differ between AS and nr-axSpA.

To our knowledge, this is the first meta-analysis systematically comparing AS with nr-axSpA. Strengths of the current study are the systematic approach in which we have chosen to include only studies that were designed to compare both disease entities, preferably in a prospective manner. Importantly, we have excluded clinical trials that included patients who were selected because of a higher activity and/or severity of their disease (which would have led to channeling bias).

This study has several limitations. First, summarizing the prevalence of peripheral and extra-articular disease manifestations was not the main objective of most of the primary studies included. This might influence the accuracy of reporting these disease manifestations (potential detection bias). Most studies did not report on how the different disease manifestations were measured. We should take into account that the prevalence of some of the disease manifestations that had ever occurred, were mainly obtained by collecting historic patient-reported information (recollection bias). However, the between-study inconsistency of most of the disease manifestations was only moderate (arthritis, enthesitis and dactylitis) or even low (uveitis, psoriasis, IBD), suggesting a significant level of agreement. Second, selection bias is another possible weakness of this meta-analysis and, more importantly, of the included primary studies. We hopefully limited the magnitude of selection bias in this meta-analysis by a thoroughly developed search strategy and other methods to increase finding accuracy (such as citation pearl growing). Within-study selection bias might be caused by different approaches to the inclusion of patients: early spondyloarthritis cohorts (such as DESIR and SPACE) possibly include different patients with shorter disease duration than are included in large observational SpA cohorts.

Furthermore, by applying the ASAS criteria for axial SpA, the prevalence of disease manifestations and characteristics might be artificially raised in nr-axSpA, because they are part of those classification criteria. The higher prevalence of uveitis in patients with AS, however, does not support this hypothesis. On the other hand, the longer mean duration of symptoms in patients with AS when compared to nr-axSpA in five of the eight included studies might confound the interpretation of the results of whether symptoms were ever present, which gives patients with AS more time to accumulate disease manifestations, and might increase the prevalence of manifestations that have ever been present. However, when we left out the studies with substantial differences in symptom duration, the results remained unchanged, except for uveitis, for which the difference no longer remained (data not shown).

## Conclusions

This meta-analysis summarized the prevalence of peripheral or extra-articular disease in patients with AS and nr-axSpA. Awareness of the prevalent nature of these disease manifestations is important in the diagnostic process, both for treatment choices and for health-related quality of life. This meta-analysis provides evidence for the largely equal nature of disease manifestations in nr-axSpA and AS, which should have consequences for research and treatment strategies.

## Additional files

Additional file 1: Table S1. PICO and search strategy. (PDF 77 kb) Additional file 2: Table S2. Estimation of bias. (PDF 91 kb)

## Abbreviations

AS: ankylosing spondylitis
ASAS: Assessment of Spondyloarthritis International Society
CENTRAL: Cochrane Central Register of Controlled Trials
CRP: C-reactive protein
DESIR: DEvenir des Spondylarthropathies Indifferenciées Récentes
ESPAC: Erciyes SPondyloArthritis Cohort
ESSG: European Spondylarthropathy Study Group
GESPIC: GErman SPondyloarthritis Inception Cohort
HLA-B27: human leukocyte antigen-B27
IBD: inflammatory bowel disease
M-H: Mantel-Haenszel
mNYc: modified New York criteria
MORE: Methodological evaluation of Observational REsearch
MRI: magnetic resonance imaging
Nr-axSpA: non-radiographic axial spondyloarthritis
PICO: participants, intervention, control and outcome
PRISMA: Preferred Reporting Items for Systematic reviews and Meta-Analyses
PsA: psoriatic arthritis
SCQM: Swiss Clinical Quality Management
SI: sacroiliac
SpA: spondyloarthritis
SPACE: SPondyloArthritis Caught Early
TNF: tumor necrosis factor
