# Supplementary material for: Prevalence of peripheral and extra-articular disease in ankylosing spondylitis versus non-radiographic axial spondyloarthritis: a meta-analysis
Source: Arthritis Res Ther. 2016 Sep 1;18(1):196. doi: 10.1186/s13075-016-1093-z (PMC5009714; doi:10.1186/s13075-016-1093-z)
Supplement: Additional file 1: Table S1. — PICO and search strategy. (PDF 77 kb) [file 13075_2016_1093_MOESM1_ESM.pdf]

PICO			
**Patients**	**"Intervention"**	**Control**	**"Outcome"**
Axial Spondyloarthritis patients	not relevant	not relevant	Extra-articular manifestations (psoriasis, IBD, uveitis)
(according to the ASAS criteria or comparable)		NB need for differentiation in radiographic and non-radiographic axSpA	Peripheral manifestations (arthritis, enthesitis, dactylitis)
			NB manifestations do not need to be the primary outcome

**Search strategy**		**No. of results**
1	exp Spondylitis, Ankylosing/	11862
2	exp Spondyloarthritis/	19312
3	spa.ti,ab.	7404
4	spondylitis.ti,ab.	12123
5	spondyloarthritis.ti,ab.	794
6	1 or 2 or 3 or 4 or 5	29896
7	(ra or di or pa).fs.	4299554
8	exp Magnetic Resonance imaging/	318500
9	exp Radiology/	25255
10	radiograph*.ti,ab.	158674
11	radiolog*.ti,ab.	180735
12	MRI.ti,ab.	145614
13	8 or 9 or 10 or 11 or 12	656937
14	6 and 13	3644
15	8 or 12	360377
16	7 or 9 or 10 or 11	4426227
17	6 and 15 and 16	1319
18	14 and 20	8
19	CASE REPORTS.pt.	1702524
20	14 not 19	2873
	Total search results	447
	Citation pearl growing	1 (extra)
Databases searched	Medline, the Cochrane Central Register of Controlled Trials (CENTRAL) and The Cochrane Library	
Date	October 1st 2015	
