# Supplementary material for: Prevalence of peripheral and extra-articular disease in ankylosing spondylitis versus non-radiographic axial spondyloarthritis: a meta-analysis
Source: Arthritis Res Ther. 2016 Sep 1;18(1):196. doi: 10.1186/s13075-016-1093-z (PMC5009714; doi:10.1186/s13075-016-1093-z)
Supplement: Additional file 2: Table S2. — Estimation of bias. (PDF 91 kb) [file 13075_2016_1093_MOESM2_ESM.pdf]

	Study							
	Esperanza	Gespic	Kiltz	SCQM	SPACE	Wallis	Desir	ESPAC
**General**								
Funding	+	+	+	+	?	+	?	?
Role of funding organization	+	?	+	+	?	?	?	?
Conflict of interest	+	+-	+	+-	+	?	+	+
Ethical approval	+	+	+	+	+	+	+	+
Aim of study: included differences between AS/nr-axSpA	+	+	+	+-	+	+	+-	+
Study design (retrospective = possible bias)	+	+	+	+	+	+	+	+
**Total**	low	low	low	low	low	low	low	low
**External validity**								
Sampling of subjects	+-	+	+	+	+-	+-	+	+
Nongeneral population based sampling method	+-	+-	+	+	+-	+-	+	+
Nongeneral population based sampling frame	+-	+-	+	+	+	+-	+	+
Assessment of sampling bias	?	?	?	?	?	?	?	?
Response rate in total sample (% missing)	+	?	+	?	+	?	+	?
Exclusion rate from the analysis	+	?	?	+	+	?	+	?
Address sampling bias	-	-	-	-	+	-	-	-
Subject flow	?	?	+	+	+	?	?	?
**Total**	low	high	low	low	low	high	low	intermed
**Internal validity**								
Source of measure of prevalence	?	?	?	+	+	?	?	?
Validation	?	?	?	+	+	?	?	?
Reliability of the estimates	+-	?	?	+	?	?	?	?
Outcome measured differently in AS and in nr-axSpA	+	+	+	+	+	+	+	+
Precision of estimate (95% CI, *p* -value)	+	+	+	+	+	+	+	+
Prevalence in AS and in nr-axSpA	+	+	+	+	+	+	+	+
**Total**	low	intermed	intermed	low	low	intermed	intermed	intermed

**For all criteria:**	
**Low risk of bias**	+
**Minor risk of bias**	+-
**Major risk of bias**	-
**Poor reporting**	?
